# Expected Free Energy Formalizes Conflict Underlying Defense in Freudian Psychoanalysis

**DOI:** 10.3389/fpsyg.2018.01264

**Published:** 2018-07-19

**Authors:** Patrick Connolly

**Affiliations:** Department of Counselling and Psychology, Hong Kong Shue Yan University, Hong Kong, Hong Kong

**Keywords:** free energy principle, conflict, psychoanalysis, defense, systems theory, neuropsychoanalysis

## Abstract

Freud's core interest in the psyche was the dynamic unconscious: that part of the psyche which is unconscious due to conflict (Freud, 1923/1961). Over the course of his career, Freud variously described conflict as an opposition to the discharge of activation (Freud, 1950), opposition to psychic activity due to the release of unpleasure (Freud, 1990/1991), opposition between the primary principle and the reality principle (Freud, 1911/1963), structural conflict between id, ego, and superego (Freud, 1923/1961), and ambivalence (Freud, 1912/1963). Besides this difficulty of the shifting description of conflict, an underlying question remained the specific shared terrain in which emotions, thoughts, intentions or wishes could come into conflict with one another (the neuronal homolog of conflict), and most especially how they may exist as quantities in opposition within that terrain. Friston's free-energy principle (FEP henceforth) connected to the work of Friston (Friston et al., 2006; Friston, 2010) has provided the potential for a powerful unifying theory in psychology, neuroscience, and related fields that has been shown to have tremendous consilience with psychoanalytic concepts (Hopkins, 2012). Hopkins (2016), drawing on a formulation by Hobson et al. (2014), suggests that conflict may be potentially quantifiable as free energy from a FEP perspective. More recently, work by Friston et al. (2017a) has framed the selection of action as a gradient descent of expected free energy under different policies of action. From this perspective, the article describes how conflict could potentially be formalized as a situation where opposing action policies have similar expected free energy, for example between actions driven by competing basic prototype emotion systems as described by Panksepp (1998). This conflict state may be avoided in the future through updating the relative precision of a particular set of prior beliefs about outcomes: this has the result of tending to favor one of the policies of action over others in future instances, a situation analogous to defense. Through acting as a constraint on the further development of the person, the defensive operation can become entrenched, and resistant to alteration. The implications that this formalization has for psychoanalysis is explored.

## Introduction

The free-energy principle (FEP henceforth) connected to the work of Friston (Friston et al., 2006; Friston, 2010) has provided the potential for a powerful unifying theory in psychology, neuroscience and related fields that has been shown to have tremendous consilience with psychoanalytic concepts (Hopkins, 2012), and may well have tremendous potential as a unifying metapsychological principle in psychoanalysis as well (Connolly, 2016). A recent paper by Hopkins (2016), drawing on a formulation by Hobson et al. (2014) has suggested how the free energy principle provides a basis for formalizing emotional conflict as complexity which places a demand (or “affective load” following Levin and Nielsen, 2009) on the capacity of the underlying generative model that predicts sensory experience to minimize that emotional complexity^1^.

Hopkins' formulation provides the seeds of a formal description of conflict in the psychoanalytic sense. He describes conflict as irresolvable complexity in the form of a complex set of simultaneous emotions that each separately motivate behavioral plans that are in conflict with one another. Hopkins (2016) draws on the example of attachment related trauma in the “strange situation” paradigm in which the disparate emotions felt by the child when their mother leaves the room - and more especially when she returns—lead to behavioral trajectories that are fundamentally in conflict with one another (e.g., anger and fear). Since there is no single action that the child can take that would simultaneously achieve predicted satisfaction for all of these conflicting trajectories, there may be persistent emotional complexity, which is Hopkins' account of trauma in this perspective.

Most importantly for this paper is how the description of conflict in his paper is founded upon the concept of free energy, and specifically the idea that distinct neural systems in the brain motivate competing plans of action which are expected to have a high cost in terms of free energy for the alternate system. In short, the Free Energy Principle (FEP) perspective suggests that a person's decision of which policy of action to follow is determined by a computation of which policy is predicted to reduce the physiological free energy (or information “surprise”) the most. From this perspective psychoanalytic conflict is presented as the state where different potential policies of action have a similar level of expected free energy, creating a subjectively unpleasant state of uncertainty of what to do. However, the present formulation of conflict and defense also necessitates a metapsychological revision of the assumptions underlying the core concepts of conflict, defense and possibly repression in line with a systems theory epistemology as spelled out in Grobbelaar (1989), which is addressed in the present article as well.

The first section will briefly describe the psychoanalytic concept of conflict, and its role in shaping defensive behavior and stable personality configurations in the person. The key problem of neurological correlates (particularly quantitatively framed correlates) is presented, including the failed explanation of psychic energy. Following this, an account of conflict from a statistical free-energy principle (henceforth FEP) perspective is explored, particularly under “expected” free energy. This formalization also suggests a route through which conflict is resolved by alteration of the relative precisions of the (beliefs about) opposing action policies. This alteration of precisions presents a means of formalizing defense, which becomes entrenched over time as a constraint on the future development of the generative model. This forms the basis for exploring the inertia of the generative model in terms of opposing the installation of certain action policies in specified situations, and also as a basis for understanding resistance in psychoanalysis as well. The implications of this particular formalization of conflict for psychoanalytic theory and practice is explored.

## Conflict in psychoanalysis

Freud's core interest in the psyche and behavior was in that part of the person that was influenced by conflict. While he noted that there were large sections of the psyche which were not necessarily involved in conflict, his stated interest was in the dynamic unconscious, which is that element of the psyche which is unconscious due to conflict (Freud, 1923/1961). Later writers such as Hartmann (1964) sought to explore the conflict-free elements of the ego, and broaden the scope of analysis. But within Freud's description of the psyche, conflict plays a central role in defining behavior, emotional and psychical experience, and personality. However, Freud's conceptions of the nature of conflict and the terrain in which it took place evolved throughout the course of his work.

Beginning with “The Project for a Scientific Psychology” (written in the late 1890s and published posthumously), Freud (1950) first outlined conflict in terms of an energy present in the nervous system (represented by a quantity he called “Qn” that “cathected” or was contained within, the neurons). He then described what he called the primary principle of the nervous system which was to discharge that activation, usually through the motor apparatus and motor activity. His first formulation of conflict was a principle that operated in opposition to the first; he called this opposing principle the secondary principle, which is the demand for discharge of activation to be inhibited, delayed, and modified in order to result in adaptive behavior. He suggested that this opposition took the form of what he termed “lateral cathexes” (discharge through laterally branching neurons) which drained the main channel of its activation toward discharge, and resulted in the activation being channeled around within a subsystem of the brain (the ψ-system which was a forerunner of the ego) in a way similar to liquid in a system of interconnecting pipes (Holt, 1962). In this first formulation conflict evidently takes place on the terrain of energies distributed in the nervous system. Due to some difficulties he encountered in developing this concept (which are described later in this paper), this conceptual centrality of energy as a zone of conflict became partly displaced by the experiences of pleasure and unpleasure, where the release of unpleasure results in opposition and suppression of mental activity that causes such unpleasurable discharge (Freud, 1990/1991), though the neurophysiological basis of this pleasure and unpleasure was never adequately described in his work. Later, Freud (1911/1963) reformulated the primary process as the pleasure-unpleasure principle, which is the tendency of the psyche to tend toward activity that produces pleasure and avoids unpleasure. In this formulation, the conflict lay between the pleasure principle and a secondary principle he now termed the reality principle, which was the need for psychic activity to generate adaptive states and behaviors by opposing the pleasure-principle. While this is formally similar to the primary and secondary process defined in “the Project,” he had then moved away from formulating these principles either in neurological or in purely energic terms, though he often tried to relate the pleasure and unpleasure to energic terms in statements made throughout his work (Connolly, 2016).

With the so-called “structural” shift toward Freud's (1923/1961) tripartite model of the psyche (the familiar id, ego, and super-ego model), rather than defining conflict in terms of the distribution and opposition of energies in the nervous system, conflict was rather stated in terms of the struggle between psychic structures (or systems): the push of the id toward satisfaction, the punitive response of the super-ego, and the ego which binds these dynamic forces. The resultant behavior, emotion or psychic experience was understood as a compromise between these forces, at times expressed in energic terms, and at others as pleasure-unpleasure and the demands of reality. This defensive compromise (which protects the person from super-ego anxiety, as well as pressure for discharge from the id) is the operation of the ego (Freud, 1923/1961). Given time, and a relatively stable environment, these compromise operations gain stability, and form the recognizable characteristics of the personality.

Beyond these descriptions of conflict, Freud also focussed on the key problem of ambivalence in the human condition, and most especially in people suffering from neurosis. In “The Dynamics of the Transference,” Freud (1912/1963) explored how analysis of neurotic symptoms often gave rise to powerful ambivalence (and resistance) in the transference, and suggested that the distorted defensive behaviors in such cases often reflected the difficult compromise between the powerful ambivalent emotions and their associated motives. Typical responses to the “strange situation” described in Ainsworth and Bell (1970) and Howe (2011), may reflect such behavioral compromises between these conflicting emotional demands (Hopkins, 2016).

## The problems of neurophysiological correlates and quantitative expression

Besides the problem of varying definitions described above, Freud's various formulations of conflict have faced more fundamental difficulties. The two key problems referred to in this paper are the problem of neurophysiological correlates of conflict, and the related problem of the quantitative terrain of conflict.

In terms of neurophysiological correlates of conflict, there is a sizeable literature outside of psychoanalysis that has attempted to discover correlates for conflict, though conflict has various definitions (and operationalization) in this literature, including decisional conflict (between equivalent alternatives), cognitive conflict (conflict between values, actions, or beliefs), informational conflict (receiving information containing contradictions), and the task of sustaining conflicting plans within consciousness, amongst others (Gray et al., 2013; Pushkarskaya et al., 2015). However, these operationalizations of conflict are not very similar to conflict in the Freudian sense, as given in the definitions at the outset of this paper, as they are mostly conscious and not apparently related to repression in any way.

Berlin and Montgomery (2017) have very recently reviewed the existing literature on neurophysiological correlates of conflict in the psychoanalytic sense. While their chapter draws together a number of interesting studies with findings that seem to have implications for conflict, few of these studies focus explicitly on conflict itself and its key neural mechanism; many of the findings relevant to conflict from this literature are more specifically about repression, suppression and dissociation, though some do have clear implications for conflict. Relevant work from this literature is from Shevrin et al. (1996) and Shevrin et al. (2013). In their approach, conflict words have been generated from transcripts of interviews with subjects, and presented subliminally and supraliminally. Measured responses were in terms of alpha power (combined amplitude and frequency measures from EEG) representing inhibitory responses toward words relevant to the conscious symptom (related to phobias in the studies). Findings appeared to show a link between unconsciously perceived conflict words acting as a prime for a greater alpha power inhibitory response toward to conflict symptom words. This supports the idea that unconscious conflicts are related to symptoms (such as phobia), and that they involve inhibitory responses relevant to symptoms, which was not found for conscious conflict words in their study.

This distinction between conscious and unconscious conflict is supported by other studies. While the role of the anterior cingulate cortex has been demonstrated in tasks involving detection and processing of conflicts related to emotion and autobiographical material (Schmeing et al., 2013), work by Dehaene et al. (2003) found that activation in the anterior cingulate cortex, which often accompanies conscious conflict monitoring tasks, was absent in subliminal conflicts. Interestingly, Anderson et al. (2004) demonstrated that conscious suppression tasks can become automated in the sense of no longer engaging conscious attention or control, after time and repetition.

More studies focus explicitly on repression than on conflict, and after a review of this field, Anderson and Hanslmayr (2014) suggest that these findings point toward the role of the lateral prefrontal cortex (PFC) in mediating inhibitory control processes, usually interacting with subcortical structures including the hippocampus and other structures encoding memories. A related set of findings (about inhibition of emotion) exist for dissociative mechanisms, such as for Depersonalization Disorder where the right dorsolateral PFC increases attention while the left PFC inhibits the amygdala and other limbic structures. This seems related to findings for both dissociative disorders as well as hypnotic states, in which prefrontal executive structures are found to interfere in voluntary and automatic processes (Berlin and Montgomery, 2017). Another interesting finding from dissociative processes is the finding of impaired connectivity in brain areas (Krystal et al., 1998). When set alongside the findings of impaired connectivity in psychosis (Schmidt et al., 2015) together with the relative lack of activation of conflict-related brain areas (including dorsolateral prefrontal cortex) in fMRI data from participants with clinical high risk for psychosis (Colibazzi, 2016, July), these findings seem to point toward the role that connectivity must play in conflict, in the sense that a minimum level of connectivity must be in place for conflict to take the form as understood in Freud's work.

While these findings have implications for psychoanalytic conflict, they do not clarify a specific mechanism for conflict that is distinct from a mechanism for repression or dissociation. In part, this lack may be due to inherent difficulties in studying conflict empirically, in the sense that the specific triggers of conflict are unique to each person. Studies focusing on conflict may use transcripts from interviews to generate conflict-related stimuli for use in the research, such as in Shevrin et al. (2013) above. Kessler et al. (2017) similarly used participant generated lists of positive and negative life events, followed by individual psychodynamic interviews based on operationalized psychodynamic diagnosis to create a list of cue sentences, used in free association and subsequent recall tasks. The resources needed for such individualized methods have undoubtedly slowed the field down. Further, the purpose of repression (and perhaps dissociation) is to avoid or reduce conflict, and so it may be problematic to measure conflict when successful repression (or dissociation) is taking place.

A second and related problem with the psychoanalytic notion of conflict is that any explanatory theory of this conflict must not only specify a shared domain or terrain between conflicting psychic processes, but also a quantitative expression of those processes such that the outcome can be understood as the difference between these quantities. While it may be correct to say that brain regions such as the prefrontal cortex, anterior cingulate cortex, limbic system, and hippocampus may be the terrain or domain of conflict (perhaps in terms of competition for neural resources or differential activation), this does not yet clarify the specific quantitative expression of the conflict. Horowitz (1977) suggested that the concept of conflict in psychoanalysis must involve a quantitative expression of some kind:

“*…the concept of conflict, deriving from the therapeutic method, has been central to all psychoanalytic clinical theory, whether the locale of that conflict was with the environment or was intrapsychic*. The dynamic and economic metapsychological viewpoints grew out of the clinical data of conflict [emphasis in original]. *How such a conflict concept would look without “quantitative” assumptions underlying it is unclear. It may be that a conflict concept would be untenable without those quantitative assumptions. In any event, no set of critiques of the economic hypotheses of analysis has presented a cogent set of alternatives in providing the underpinning for the dynamic viewpoints” (Horowitz*, *1977**, p. 563)*.

We should agree with Horowitz that conflict is untenable without quantity. For conflict to take place, not only should two phenomena exist within a shared terrain in which they can interact, but they should also be able to exert some form of influence upon one another, which is meaningless if not theoretically quantifiable (Swanson, 1977).

## The failed solution of psychic energy

Freud's key attempt to provide this quantitative account of conflict lay in his theories of psychic energy, and the principle of inertia which was proposed as regulating those energies. This is most pronounced with regard to the original formulation of conflict (described at the outset of this paper) which is conflict between the primary and secondary processes as defined in “The Project for a Scientific Psychology” (Freud, 1950). As described above, Freud proposed this conflict as a contest between quantities of Qn in the nerves: the result was either that the energy was retained in the ψ-system or progressed toward motor discharge, or some compromise of the two. The result was essentially determined by the levels of the quantities at play.

However, this explanation of cathexes of energy within neurons failed. The energic theory has been widely critiqued by a number of authors, and a detailed review of this debate that unfolded over several decades can be found in Connolly (2016). The most common critique has been the lack of any sound empirical evidence from brain science of the energic processes as described in “The Project” (Basch, 1976; Swanson, 1977; Zepf, 2010), and what we now know about the nervous system which is that action potentials vary in terms of frequency, but not in terms of intensity or strength (Pribram and Gill, 1976). Rapaport (1960) also outlined a familiar argument that the energic processes can't be observed directly in the clinical situation. However, the key failure that Freud himself was aware of, and which led him to eventually abandon “The Project” was irresolvable internal contradiction in the proposed model. This was because his description of the higher functions of the psyche (e.g., consciousness, memory, attention, and others) relied on a linear progression of stimulus energy from the sense organs and sensory stimulus, through the ψ-system, the system of conscious experience (or ω-system) and on to motor discharge. However, once Freud tried show how this progression of energy through these systems actually produced the phenomenology of attention, consciousness, and memory, he was forced to add constructs, revise, and rework, until he eventually radically changed the entire structure in the final pages of the collected document, and never elaborated further on this change, but instead tried to suppress the text after that.

In later work, while Freud appeared to back away from further theorizing about the quantities of Qn, he retained concepts of energy and cathexis, and in “The Interpretation of Dreams,” Freud (1900/1991) viewed conflict in terms of stimulus energy moving “forward” through the psychic apparatus (toward motor discharge) being opposed by inhibition from the preconscious gate to prevent unpleasurable discharge. At the same time a regressive movement of excitation backwards through the apparatus took place (usually in sleep or hallucination), “powered” in a sense both through inhibition, as well as by a “pull” of powerful sensory memories (Connolly, 2016). Regarding this latter text, it is important to note that while Freud was still talking about a contest between theoretically quantifiable amounts of energy, he had moved further away from specifying the physiological expression of the energy, and therefore further away from specifying the physiological terrain in which conflict between these energies could take place. Though Freud (1920/1955) developed his ideas about psychic energy further in “Beyond the Pleasure Principle,” he never escaped internal contradictions of his energic theory, nor came closer to clarifying its physiological substrate (Basch, 1976; Zepf, 2010), despite continuing to use concepts of cathexis, discharge, and libido throughout his career (Holt, 1962; Connolly, 2016).

Despite the failure of the energic theory to provide a working quantitative account of conflict within psychoanalysis, the problem has remained as a troubled foundation of the field until recently.

## Conflict within a free energy principle (FEP) perspective

Friston's (2010) free energy principle has become of rapidly growing interest to psychoanalysis due to significant formal similarities between FEP and several assumptions within psychoanalysis, including the primary principle of mental functioning (Carhart-Harris and Friston, 2010), unconsciousness and motivation (Hopkins, 2012), emotional complexity in attachment (Hopkins, 2015), wish fulfillment within dreaming (Hopkins, 2016), and the energic theory within psychoanalysis (Connolly, 2016). The FEP also has the potential to offer a quantitative basis for a formulation of conflict as well (Hopkins, 2016), which could solve the problem of a quantitative expression of energy and conflict as well as its neurophysiological substrate or terrain.

Essentially, the FEP proposes that the physical structure of all self-sustaining and adaptive creatures encodes a model of the sensory inputs emerging from their environment. The FEP then states that living systems must then, either implicitly or explicitly, minimize their variational free energy. What is meant by variational free energy here is a quantity of informational surprise or prediction error, which is the difference between the sensory states predicted by the model, and those that are actually received. This leads to living systems avoiding surprising or highly improbable states. This is consistent with a principle from physics known as Hamilton's principle of least action (determining the path of lowest value), cast in terms of information theory. Mathematically, negative surprise is the same as (log) Bayesian model evidence (Friston, 2009). This means that creatures (or people) that minimize their free energy also maximize the evidence for their model of the world, or in other words, are self-evidencing (Hohwy, 2016). Importantly for the present argument, free energy can be minimized (and model evidence maximized) by one of two routes: either by an updating of Bayesian beliefs encoded by the generative model of the organism, or by taking an action which alters the sensory inputs of the organism in such a way that the surprise (or prediction error) is reduced.

A core significance of the FEP for psychoanalytic theory, is that it offers a potentially quantifiable formalization of Freud's concept of psychic energy. However, FE is not a physical energy, but an information theoretic concept; it does not quantify a thermodynamic energy, but rather quantifies a form of information present in the system: in this case the biological organism (Friston, 2010). However, the FEP can still play a very similar role in psychoanalytic theory to that played by the energic theory (as a formalization of the core motivator and organizational principle of activity in the person and their mind), though it may necessitate the incorporation of a systems theory epistemology to adequately do so (Connolly, 2016; Connolly and van Deventer, 2017). Most importantly for the present paper, it provides a basis for understanding how psychic processes can be quantitatively expressed.

However, as indicated earlier, beyond this requirement of a quantitative expression of conflict, there is the requirement of a shared terrain in which these quantities can come to “oppose” one another. A FEP perspective supplies this formalization of the shared terrain, though noting both the scale-free nature of the FEP in the physiological organization of the organism, and also the complexity and differentiation within the organism. Essentially, we might think of the overall organism as being constituted of a massive complexity of subsystems, that extend from sub-cellular components or organelles (e.g., dendrites or mitochondria), through cells (e.g., neurons or others), tissues, organs, systems and even higher levels of recursion. The important insight here is that each of these subsystems obey the FEP, each tries to minimize its free energy. In other words, all structures in the organism that have an identifiable boundary condition (a Markov blanket) minimize their free energy. Further, the total free energy present in the whole organism is understood as the sum of the FE present in each of the constituent subsystems (Friston et al., 2015a). This has the implication that while changes in the activity of the whole system (i.e., organism) reduce the overall FE of the system, they may at the same time, *raise* the FE of specific subsystems. An example might be the organism's response to muscles enduring sustained strain; while the organism's overall FE might be lowered by the activation of the sympathetic nervous system which brings needed oxygen and nutrients to (and removes waste products from) the strained muscle tissue, the tissues of the heart itself are pushed further from equilibrium, and may experience a relative increase in FE which is attempting (though initially failing) to drive the overall system in the opposite direction, to reduce blood flow. This formulation now provides us a basis for understanding how a theoretically quantifiable form of “conflict” (in a broad sense) can take place in the terrain of information exchange between subsystems within the biological organism.

However, this definition of conflict in the organism is broader than that implied by psychoanalysis, as this form of conflict is ubiquitous to every level of scale in the organism, whereas we might say that psychoanalytic conflict occurs at a particular level of organization in the organism. We may even find a range of similarly broad conflict phenomena at a psychological level of interest in the organism which still do not precisely equate with the psychoanalytic concept of conflict. There are a number of such potential examples.

With regard to perception, Hopkins (2012) describes a formal similarity of this form of conflict with an artificially induced binocular rivalry paradigm, where the right and left eyes are given different objects in their field of view (e.g., a face and a house). The result is that perception oscillates between seeing a house for some time, then a face for some time, and back to a house, and so on. The neural structures that encode a house image are in competition with those that encode a face image to activate a dominant perceptual inference. Should the house image become the first dominant inference, then the sensory stimuli that are associated with the face persist as surprise (prediction error); the persistent surprise feeds upwards to the higher levels again which shifts the dominant inference to that of the face, where the stimuli from the house now feed upwards as prediction error, and so on. Hopkins (2012) suggests a consilience between this process and that of the psychoanalytic unconscious where the dominating inference renders the conflicting stimuli unconscious (usually on a more enduring basis), though the surprise associated with the suppressed stimuli still motivates automatized unconscious behaviors, though they cannot become conscious inference. This scheme is easily extended to explain the repression of sexual excitement for example, where interoceptive stimuli emerging from sexual excitement remain suppressed by a more dominant inference that doesn't integrate the stimuli, which nonetheless can activate automatized behaviors. This mechanism of the unconscious is returned to later in the paper.

Another example of this type of conflict in inference at a “psychological” level of interest (close to, but perhaps not the same as psychoanalytic conflict), refers to the perception of emotion, or the occurrence of feeling two apparently contradictory emotions at the same time, which we might encapsulate in the statement “I don't know what to feel, nervous or excited.” Examples of such conflicting emotion inference from experimental science might include the studies of misattribution of arousal (Dutton and Aron, 1974; White et al., 1981) where distinct sources of arousal may nonetheless activate a dominant inference, for example where arousal due to the effort and nervousness from crossing a bridge appears to increase perceptions of attractiveness of a research confederate. This highlights a potentially important aspect of active inference in exchange with the world—and one's body. Namely, one has to infer the causes of all sorts of sensations; including proprioceptive and interoceptive (i.e., motor and autonomic) signals. In other words, we have to find explanations that account for all our sensations and select the most plausible hypothesis that best explains them. This means that interoceptive inference about the state of my body contextualizes exteroceptive sensory cues concerning “where I am” and “what I am doing.” This means that sensations of autonomic arousal have to be explained (away); thereby leading to the hypothesis or explanation that “I am currently in a particular emotional state.”

While the researchers in the above attribution studies have not explicitly connected their experimental findings either with psychoanalytic conflict literature or with active inference, they may nonetheless demonstrate such competition for awareness between conflicting emotional signals that may potentially be explained from a FEP perspective in a similar way as the binocular rivalry findings are explained in Hopkins (2012), in that one inference tends to dominate at a time. As suggested earlier however, while these examples occur at the level of interest in psychology more broadly, they may not reflect examples at a psychoanalytic level of explanation, as they primarily reflect conflicts in perception. From the earliest phases of Freud's work, psychoanalytic conflict has been linked to the inhibition of *action*, typically through inhibition of the flow of energy toward discharge through the motor apparatus (Freud, 1950; Breuer and Freud, 1985/2004). Expected free energy offers a formal description of action selection (Friston et al., 2017a) that offers potential for such a formalization of psychoanalytic conflict.

## Expected free energy and selection of action

To understand psychoanalytic conflict—from the point of view of FE minimization—it is necessary to consider a slightly nuanced aspect of the FEP; namely, active inference and planning of action, or *expected* free energy. Expected free energy refers to the predicted level of free energy after a course of action is taken. A course of action is referred to here as a policy. A priori, the probability of selecting a particular policy decreases with the free energy expected under that policy. To refer to another example found in Hopkins (2012), a person who is experiencing surprise in the sense of interoceptive signals of dehydration or thirst, may seek a glass of water, as that course of action will have the lowest expected free energy of the various possible courses of action. In this case a generative model specifies the expected free energy following alternative courses of actions (i.e., policies) and the policy that leads to the least surprising outcomes is selected (i.e., “my thirst will be quenched”).

To understand the nature of expected free energy, one can decompose it in various ways. For the purposes of the current argument, one can think of expected free energy as comprising epistemic and pragmatic parts (Friston et al., 2015b). The epistemic part tries to resolve uncertainty by taking actions with high information gain—that resolve ambiguity about the state of the world (e.g., Kapur, 2003; Itti and Baldi, 2009; Mirza et al., 2016). The pragmatic part simply reflects the prior beliefs (e.g., about drinking water) or preferences ingrained in a generative model through prior experience (or perhaps epigenetics). Friston et al. (2017a) suggest that any organism that has prior beliefs about its behavior must believe it will minimize expected free energy or, more simply, resolve uncertainty under prior beliefs about what will happen to it.

**Figure 1:**
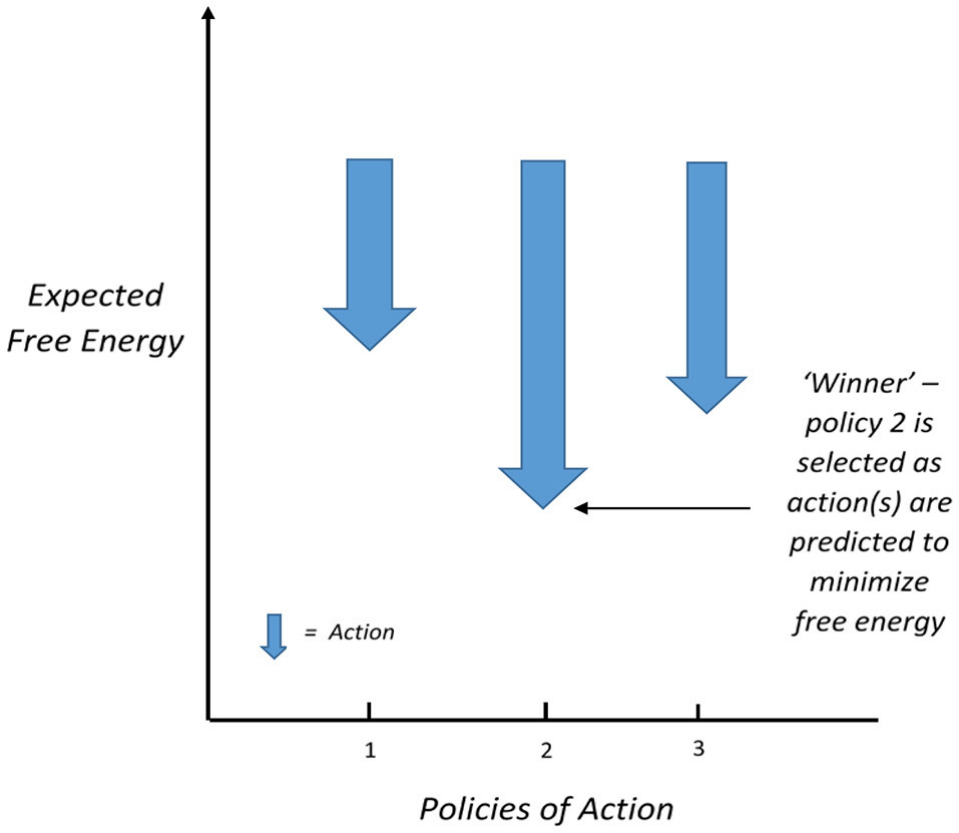
According to Friston et al. (2017a), the nervous system calculates the expected free energy of actions under different policies of action, and those with the lowest expected free energy gain dominance (after Connolly, 2017, with permission from the copyright holder).

Friston et al. (2017a) suggest that it works as follows: where sensory inputs generate surprise at lower levels of a hierarchy of perceptual inference, they trigger potential action plans at higher levels. Those action plans can be evaluated in terms of the expected free energy, informed by expectations encoded by a hierarchical generative model. This is especially interesting if one considers organisms (particularly human beings) that entertain different outcomes under different choices (or “policies” of action) at the same time. This allows for selecting actions that have the smallest expected free energy (Friston et al., 2015b). The researchers contend that this approach resolves the difficult problem of selection of action into an “easy” inference problem. This selection of action policies is usually expressed as a softmax function where the probability of an action is equal to the exponential of negative expected free energy (normalized so that the sum of probabilities is one; Friston et al., 2017a) and is represented in Figure 1 below.

The authors demonstrate how this proposed process is neuronally plausible:

“*This reflects a process theory which associates the expected probability of a state with the probability of a neuron (or population of neurons) firing and the logarithm of this probability with postsynaptic membrane potential. In this approach, post-synaptic depolarization caused by afferent input can be interpreted as free energy gradients (or state prediction errors) that are linear mixtures of firing rates in other neurons (or populations). These prediction errors drive changes in membrane potential and subsequent firing rates (Friston et al., 2017a).”* (Connolly, 2017).

For those less familiar with the FEP, this process theory provides a concrete understanding of what the quantity of free energy is (in terms of the nervous system at least), which is the level of influence that the activity of neurons or populations of neurons have on the rest of the system.

Besides suggesting the neuronal plausibility of this approach to action selection, Friston et al. (2017a) propose a potential functional anatomy of the process as follows:

“*Sensory evidence is accumulated to optimize expectations about the current state of the world, which are constrained by expectations of past and future states. This corresponds to state estimation under each policy the agent entertains. The quality of each policy is evaluated in the ventral prefrontal cortex, possibly in combination with ventral striatum (van der Meer et al., 2012), in terms of its expected free energy. This evaluation and the ensuing policy selection rest on expectations about future states. Note that the explicit encoding of future states lends this scheme the ability to plan and explore. After the free energy of each policy has been evaluated, it is used to predict the subsequent hidden state through Bayesian model averaging (over policies). This enables an action to be selected that is most likely to realize the predicted outcome. Once an action has been selected, it generates a new observation, and the cycle begins again (p. 19).”*

While the authors' proposed functional anatomy is not yet supported with specific empirical proof, it is nonetheless consistent with what is generally accepted about the functional anatomy of the brain, and is presented by the authors as a possible anatomy rather than a proposed model. Its purpose is to offer support for the proposed formulation of action selection through expected free energy by describing how it might be reflected in the functioning of the brain.

Recent work on canonical microcircuits (Bastos et al., 2012) have also supported the idea that the layers of cortical columns (including functional separation of higher and lower levels of neurons and interneurons) show this form of hierarchical organization of neurons which is able to sustain the computations involved in estimating expected free energy of this kind. In this work, afferent projections from lower-order areas feedforward prediction errors which excite expectancies encoded by populations of neurons connected at that higher level; these offer inhibitory feedback connections, through stimulating inhibitory interneurons in the lower-order layers. This computational architecture allows for higher-order expectations that strongly increase free energy to more strongly inhibit lower-order stimuli that give rise to them, allowing for the phenomenology described above, which involve policies with high expected free energy to be inhibited in favor of policies which reduce the expected free energy.

**Figure 2:**
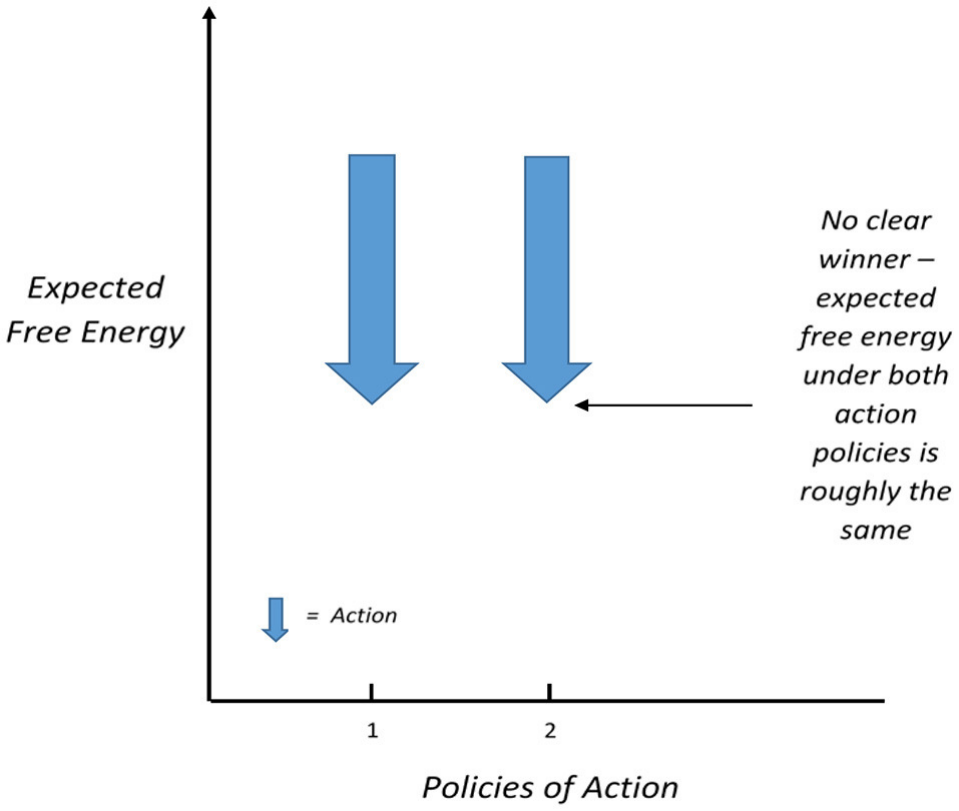
A mathematical or computational homolog of psychoanalytic conflict (after Connolly, 2017, with permission from the copyright holder).

The central importance that this formulation has for the present paper, is that it offers a great opportunity to provide a formal description of conflict from a FEP perspective. Conflict, from a FEP perspective can be formalized as follows: if every action has roughly the same expected free energy there is no clear winner—and the probability or belief distribution over alternative ways forward becomes uncertain; in other words, beliefs about what I am doing have a low precision. This is the mathematical homolog of conflict; namely, a loss of precision or confidence in what to do next, and is represented in Figure 2 below.

With this model or formalism in mind, we can now see how difficult it must be for a person who entertains different actions or policies that each only satisfy one of a number of precise prior beliefs. This is the form of irreducible uncertainty posed by conflict problems, and is consilient with the description of conflict within the psychoanalytic literature reviewed earlier.

## Conflict in the strange situation

**Figure 3:**
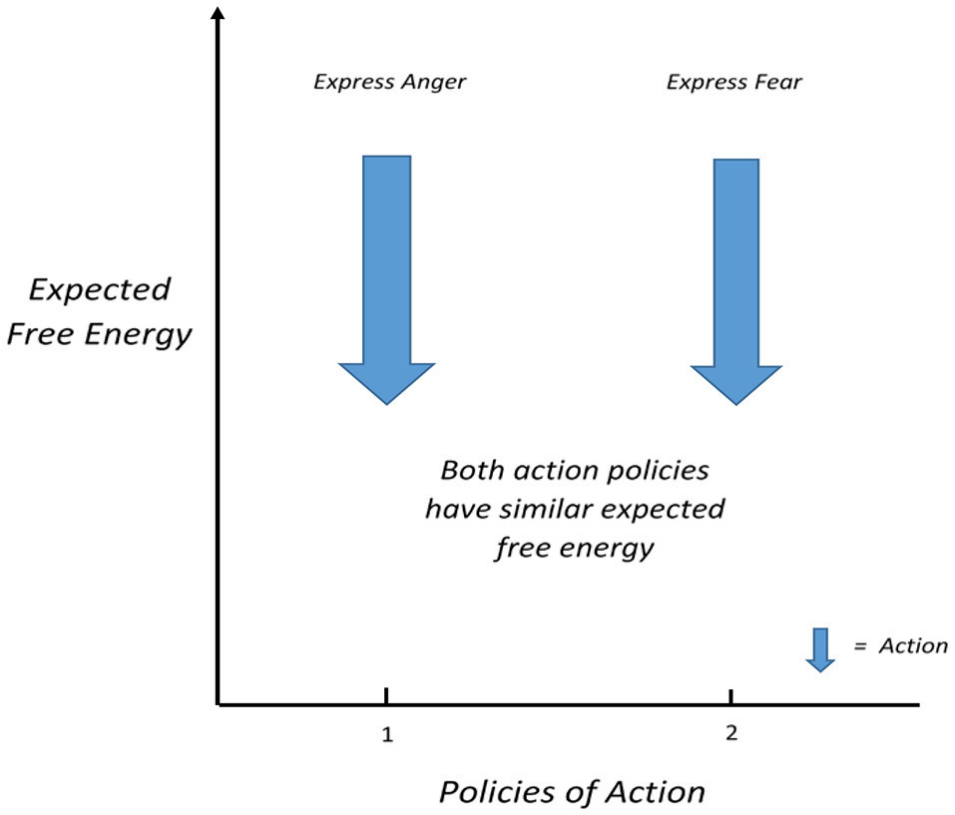
Conflict between similar levels of expected free energy of actions motivated by prototype emotion systems of FEAR and RAGE in the strange situation (after Connolly, 2017, with permission from the copyright holder).

We may now apply this scheme to the formulation outlined in Hopkins (2016). The prototype emotion systems described by Panksepp (1998) can be understood as functional subsystems of the nervous system, ones that even have visually identifiable boundary conditions, in some respects. As described in Hopkins (2015) the strange situation simultaneously gives rise to activation of a number of these prototype emotions systems. Following the formulation in Hopkins (2012), each of these will give rise to a (best-guess) belief that a particular behavior will satisfy demand from the prototype emotion system and reduce the surprise associated with it. So, while it may be that striking the mother may satisfy the RAGE system, it is also likely that this will increase the chance of losing her, which would increase the free energy (surprise) associated with the FEAR system. In terms of active inference, what is being said here is that the particular structure of the RAGE and FEAR neural systems encodes particular prior beliefs about outcomes that can be realized by different courses of action. The conundrum here is that all available courses of action lead to violation of prior beliefs (i.e., an increase in surprise or free energy) in at least one dimension (i.e., the prototype emotions; Panksepp et al., 1984). This situation (represented in Figure 3) may make it difficult to reduce the FE of both of these systems, leading to persistent distress (trauma, in Hopkins' view), such as that found in the “insecurely” attached pattern of response. In this way, the insecurely attached child in the strange situation may cycle between feelings of fear and rage, much as perception cycles in the binocular rivalry paradigm described in Hopkins (2012), where the stimuli related to the currently non-dominant inference persist as surprise (i.e., unresolved prediction error) that pushes the alternative inference into dominance, and back again, and so on.

It is important to note that the example drawn from Hopkins (2016) here is an example of an application of the formal definition of conflict offered in this paper which is the situation of competing policies of action with relatively similar free energy. Theoretically, other subsystems of the brain, other than the prototype emotion systems could demonstrate this form of conflict, provided they are motivating competing action plans, and produce meaningful increases in free energy. Nonetheless, this example underlines the importance of the prototype emotion systems delineated by Panksepp (1998), and their central significance in a psychoanalytically-informed description of human consciousness and behavior as described in Solms and Turnbull (2003). Not only are these systems potent sources of free energy in the organization of the brain, but they also create the conditions for significant conflict in action selection, and explain the intensity of agitation and emotional distress caused by the persistent FE from activation not resolved by the currently dominant policy (or state estimation from predicted actions; Hopkins, 2016).

A detailed computational model of the interaction between caregiver and child that simulates the emergence of an attachment pattern in the child has recently been published by Cittern et al. (2018). In their model a Bayesian approach based on active inference (based on the FEP) is deployed within a game theoretical framework where a child agent has three available actions, “seek,” “guarded seek,” or “avoid.” In return, a caregiving agent may be “highly responsive,” “inconsistently responsive,” and consistently “unresponsive,” expressed in terms of “attend” or “ignore” behavior. This model simulates a situation in which the interoceptive states of the child following “attend” or “ignore” behavior may steadily result in one of the typical organized attachment patterns of “secure,” “avoidant,” or “ambivalent.” This simulation provides some support for the key propositions of attachment theory which is that the pattern of caregiving behavior shapes the subsequent attachment pattern of the child (Bowlby, 1969, 1973). Where affective communication errors (ACEs or cues that are misleading with regard to the subsequent behavior) are added to the model in the form of an exteroceptive cue before the “attend” or “ignore” behavior, they add further explanatory value. High levels of ACEs before inconsistent responding produced an ambivalent attachment pattern, while inconsistent cues before consistently distressing responding produced a disorganized attachment model. This is in line with research which has shown that affective communication errors are associated with both ambivalent and disorganized attachment patterns (Bronfman et al., 1999; Safyer, 2013). The observation that these models produce responses that are in line with what is expected from previous theory and research on attachment, is offered as strong support for a FEP based computational model of the child's responses to a caregiver (Cittern et al., 2018). With regard to the formulation being offered in this paper, this work supports the grounding of a process of conflict within a FEP-based model, that may be organized by interactional process as suggested by Hopkins (2016).

The present formulation of conflict also provides the basis for generating a reformulation of a psychoanalytic explanation for the development of an unconscious due to defense, through a description of how the brain processes and overcomes this conflict state through an alteration of the relative “precisions” of the demands associated with these emotion systems, described next.

## Defense as altered precisions

Referring back to the description of the expected free energy of action policies in Friston et al. (2017a), prior beliefs about outcomes (that underwrite pragmatic value) could themselves be inferred. This offers an insight into how conflict could be overcome or resolved: namely by assigning greater precision to a particular set of prior beliefs about outcomes to resolve ambiguity in situations of conflict. The term “precision” here refers to the range of variation allowable within incoming information; higher precision means that even minor variations in stimulus values may generate error, whereas lower precision means that incoming information can vary a lot more before generating surprise. We might use an analogy found in Peterfreund and Schwartz (1971) about a thermostat: if a thermostat only allows for a variation of two degrees on either side of the optimum temperature before activating an air conditioner, it will be activated far more easily or often than a thermostat that allows variation of four degrees on either side. The narrower range of variation allowed by the thermostat (two degrees) is similar to a situation of higher precision, where more minor variations can generate strong error.

In the above example, precision can be regarded as the sensitivity of posterior beliefs to some form of evidence; in other words, the confidence we ascribe to evidence. Exactly the same interpretation applies to the precision of beliefs about discrete outcomes; say, for example a number of competing or conflicting policies. In economics, the precision corresponds to the sensitivity parameter of a softmax function; also known as inverse temperature. In short, a precise belief distribution means that there is one clear winner and we are confident about some state of affairs. In what follows, we will start by considering the precision of beliefs about courses of action; i.e., “what am I doing”—and then drill down to the prior beliefs about outcomes that underwrite policy or action selection. The balancing of different prior preferences depends upon the precision of these preferences, emphasizing one sort of outcome over another.

Applying this to psychoanalytic conflict again, by assigning a higher precision to one of the conflicting alternatives, ambiguity or uncertainty (about policies) can be resolved because one course of action reduces expected free energy more potently than all alternative choices (there is now a clear winner, in the economics sense described above). Intuitively, this is essentially the same as assigning a greater “importance” to minimizing the surprise of one of the subsystems as opposed to the others, through altering the precision of prior preferences about outcomes encoded by these subsystems. Referring back to the previous example of prototype emotions, this would be equivalent to assigning greater importance to satisfying either the RAGE or FEAR prototype emotions, thus tipping the balance of expected free energy in favor of one or the other.

## The formation of an unconscious

Significantly, the consequence of this change, is that the other non-dominant action plan is now no longer determining the action plan that becomes represented in conscious experience and is acted upon. However, the prediction error associated with this now non-dominant alternative is not entirely removed either—this persisting error may potentially be reflected in the apparently “unconscious” agitation or intensity that appears to accompany conflict in the clinical situation, even when the person is only aware of the dominant inference regarding their own mental states.

This formulation of overcoming conflict seems consilient with Freud's (1915/1963) claim about the conflict that underlies repression:

“*Let us confine ourselves to the clinical experience we meet with in the practice of psychoanalysis. We then see that the satisfaction of an instinct under repression is quite possible; further, that in every instance such a satisfaction is pleasurable in itself, but is irreconcilable with other claims and purposes; it therefore causes pleasure in one part of the mind and ‘pain’ in another. We see then that it is a condition of repression that the element of avoiding ‘pain’ shall have acquired more strength than the pleasure of gratification (p. 105).”*

Stated in the language of the current paper, the pleasure of gratification of an instinct is here being understood as the “pleasure” of reducing the FE of a subsystem (a “part of the mind” as Freud suggests, in the current example, one of the prototype emotion systems). However, despite this important consilience with Freud's perception of repression, the mechanism of defense described here also has some important differences from the accepted description of conflict and repression in Freud's work. Specifically, what is missing from this description of the dynamic unconscious is almost the entire description from Freud's (1923/1961) structural theory, which is the role of a repressive action from the ego to avoid anxiety. This might lead one to suggest that the unconsciousness described above (due to not activating a dominant prediction) is not the same thing as that described by Freud. This is correct, it is not the same. The formulation of conflict and unconsciousness being presented here demand a different metapsychological assumption from that articulated in Freud's work.

Specifically, what is needed to incorporate a FEP-inspired description of conflict, repression and the unconscious within Freudian metapsychology is a systems-based epistemology which suggests that all the key mental processes of interest to psychoanalysis must be located within a hierarchy of organization, and must themselves be founded upon and constrained by processes at lower levels of the hierarchy (Connolly and van Deventer, 2017). This was expressed best in Grobbelaar (1989) as follows:

“*As it stands now, Freud's formulation of the process of censorship defines it as an ad hoc defensive maneuver by one system, the ego, against another system, the unconscious, to stop dangerous elements (dangerous to the organization of the ego) from entering the ego. One should rather formulate from the bottom to the top, that is, in a theoretical sense. One should begin by defining the inherent qualities in the lower-order elements which … make it impossible for them to be taken up in a higher order system … (p. 142).”*

He elaborated on this further:

“* … the principles determining the perception of thoughts will be inherent in the thoughts themselves. Stated differently, if the organization of the ideational domain does not allow for the representation of certain ideas, thoughts or memories, then they cannot become conscious (p. 139–140).”*

The incredible value that the FEP (and more specifically the current formulation of expected free energy of competing policies of action) can have for psychoanalysis is that it offers precisely such an explanation that offers a hierarchical description of the processes and also implies that defense (or repression) as a process must have its origin in process at a lower level of organization than consciousness, as described next.

To view precisions in terms of a hierarchical arrangement of functions in the brain, the precisions associated with a particular level of functional hierarchy are essentially determined by activity and structure at higher levels of hierarchy than that level at which the conflict takes place. In one regard, this refers to the range of possible states that can be encoded by activity at the higher levels. In terms of complexity of organization, it means that lower levels of complexity of encoding at the superordinate level result in higher levels of precision associated with surprise from subordinate levels (Mathys, personal communication, 14 July 2017). With regard to resolving conflict, this means that the generative model at a higher order comes to encode a more limited set of possible states with regard to one of the conflicting neural systems.

This is also important with regard to conscious experience. If we suggest as Hobson et al. (2014) have, that consciousness might refer to the process of inference at higher levels of brain hierarchies, then this might imply that the person in our example would likely only consciously experience the fear-related response, and be less aware of anger in their response. Note that it is possible that “automated” motor responses to anger, which may be triggered at levels of processing far below consciousness, such as forming a fist or clenching teeth, may nonetheless persist in the person's behavior. However, we might expect that they are usually not attended to by the person, though it is these behaviors which may typically be pointed out to a client by a psychoanalytically-oriented psychotherapist.

In this hierarchical setting, it is possible that policies unfold at different hierarchical levels, where the precision of prior preferences—that underwrite expected free energy—are supplied by supraordinate levels. In what follows, I will use precision as a shorthand for the precision of various prior preferences that determine policy selection at each and every level of inference. This comfortably accommodates the above phenomenology. For example, I can select low level (automatic and autonomic) policies that entail “fist clenching” and yet ignore this evidence that I am “angry” at a higher level of inference, if there is a more plausible (or “important”) explanation or policy at hand (e.g., “I must do this to avoid being frightened”). In short, the precision-afforded prior preferences throughout the hierarchy play a crucial role in contextualizing the evidence for my current narrative and course of action—that will necessarily entail competition and ambiguity at each and every level^2^.

The present formulation offers a potential formalization of defense related to conflict. Here, defense must refer to constraint reflected in the encoding at a level of functional hierarchy superordinate to that of action selection described earlier that results in an imperative to favor one policy of action over others. Referring to the above example, this could mean favoring a policy of action driven by the “FEAR” system rather than that of “RAGE.” The result is that in future situations similar to that which triggered the conflict, we might expect the child from the “strange situation” example to be more likely to show a fear-related response and cling to the mother, and less likely to show an angry response.

## The entrenchment and progressive complexity of defense mechanisms through development

This formalization of defense has implications for the further development of the person. The development of a person is characterized as the emergence of successively higher levels of hierarchical organization in the brain, as well as the progressive increase of complexity within those levels.

Further, following Grobbelaar (1989), and Connolly and van Deventer (2017), we could state that the constraints that operate at one level of a hierarchy must be reflected at higher levels of recursion. This means that the more “rigidly” encoded precisions of the defense must act as a constraint to the further development of the organism, including through hierarchically superordinate levels. This is formally similar to Freud (1912/1963) statement in “The Dynamics of the Transference”:

“*Now our experience has shown that of these feelings which determine the capacity to love only a part has undergone full psychical development; this part is directed toward reality and can be made use of by the conscious personality, of which it forms a part. The other part of these libidinal impulses has been held up in development, withheld from the conscious personality and from reality, and … may remain completely buried in the unconscious so that the conscious personality is unaware of its existence (p. 106).”*

This developmental aspect of defense is a critical element of a psychoanalytic view of the person. It is a common assumption within a psychoanalytic approach that a wide range of diverse adult behaviors are nonetheless thematically related to one another as being underpinned by a common defensive operation which has its origin in a critical event or situation from early childhood (Greenson, 1967). Returning to our example, we might find that the child who formed an imperative toward action policies related to fear rather than anger in situations that activate both (and that imperative has constrained further development) may as an adult exhibit a general inhibition of angry responses, and privileging of fearful behaviors in situations that call for both. For example, in a future adult relationship, when the person's partner arrives hours late for a meeting with little explanation or empathy, the person may appear to excessively seek reassurance rather than (consciously) expressing anger. Similarly, they may usually advise friends to behave in a placatory manner instead of an angry one when feeling mistreated by their partners. They may feel uncomfortable when observing someone expressing anger at their partner over perceived neglect, and try to avoid being exposed to situations where they might observe this behavior. Though these behaviors occur in different settings and situations, and reflect a complexity of influences, the present formulation attempts to show that they may indeed be traceable to a constraint on the developing hierarchical structure of the generative model that emerged at an early age, and became foundational to an emerging structure of perceptual (and action) prediction.

This progressive development of complexity of behavior and psychic activity associated with the defensive encoding of precisions is connected with clinical theory in psychoanalysis where it is proposed that the unconscious material comes into association with other elements of structure in the psyche, resulting in a diversification of expression of the related defense. Freud (1915/1963) describes this process from a clinical perspective:

“… repression proper [emphasis in original], *concerns mental derivatives of the repressed instinct-presentation, or such trains of thought as, originating elsewhere, have come into associative connection with it. …We have to consider… the attraction exercised by what was originally repressed upon everything with which it can establish a connection. Probably the tendency to repression would fail of its purpose if these forces did not cooperate, if there were not something previously repressed ready to assimilate that which is rejected from consciousness. …we are inclined to … forget too readily that repression does not hinder the instinctual presentation from continuing to exist in the unconscious and from organizing itself further, putting forth derivatives and instituting connections (p. 106).”*

The above quote focuses on the progressive association of what is repressed with other elements of the psyche rather than the constraint related to the defense. In the current formulation, we might focus on the other side of the coin which is the increased precision of the now-dominant action policy, which must come to be applied to all new situations which trigger the previously conflicting state, such that the predictions associated with the dominant defensive response become ever more elaborated, through the ordinary development of the individual.

## Inertia and therapeutic resistance

From the perspective of active inference, the tendency to increase the complexity of generative modeling within a creature's comfort zone can be understood in terms of free energy minimization: in the same way that expected free energy can be divided into epistemic and pragmatic parts, the free energy itself can be expressed as accuracy minus complexity (Hopkins, 2016). This means that as the generative model is optimized (i.e., learned through experience), it will try to provide more and more accurate explanations for its sensations. This will necessarily incur a complexity cost. Provided the accuracy increases—with learning—to a greater extent than the complexity, free energy will continue to decrease. This accuracy of the model is also dependent in part on the relative plasticity of the environment, such that the person can shape the environment in such a way that the generative model is accurate. This means that if a creature can find and construct its own “econiche” (an environment that fits and sustains the predictions of their model of the world), that generative model will increase its complexity only up until a point that there is no further gain (in terms of accuracy). Beyond this point, the phenomenon of (statistical) overfitting emerges. This corresponds to a failure to generalize the model to slight changes in the data, which means our model is no longer optimal to explain the normal levels of variation of data in our econiche. In this case, the generative model then appears to resist further change, provided the environment adequately supports the model as it is.

This process is important to psychoanalysis as it could explain the tendency to maintain a particular defensive constraint in the encoding of precisions of prior preferences that shape expected free energy and ensuing policies. These preferred outcomes specify states that become attractor states; namely; states to which the system is attracted; thereby maintaining its own organization and remaining within particular boundary parameters. The notion of self-maintenance and attractor states speaks directly to the premise of the free energy formulation—in the sense that the raison d'être for minimizing free energy is to establish and maintain experienced states within some attracting set; specified largely by prior beliefs (Friston, 2013). This theme emerges at many levels in self-organization; ranging from self-assembly in computational chemistry and molecular biology (Cademartiri et al., 2012; Friston et al., 2015a), through to autopoiesis (self-creation) in biological self-organization (Maturana and Varela, 1980; Thompson and Varela, 2001). The key point here is that if the priors that anchor the choice of action policies (to resolve conflict) become too entrenched, a particular, self-fulfilling, self-sustaining pattern of behavior emerges. Indeed, if this pattern involves placatory or reassuring behavior in the face of apparently devaluing behavior from others, one can imagine a particular personality phenotype (or ego-structure) that avoids aggressive behaviors within relationships altogether, to the extent that this behavior is successful and sustainable in meeting the expectations of the generative model.

The argument here is that “inertia” may reflect an entrenchment of prior beliefs that are sculpted by the imperative to avoid conflict and, in epistemic terms, the implicit uncertainty. Again, we see the imperative of reducing expected free energy or uncertainty in driving both behavior and the prior beliefs that underwrite that behavior. What this means regarding the present formulation is that as the generative model of the person continues to develop in complexity and hierarchical organization, so the constraint of precisions regarding action policies related to conflict come to be proportionally reflected in the generative model as well (though noting that it is also depending on the plasticity of the environmental niche as well). This means that the free energy cost of altering the precision of preferences that underwrite policy selection also increases with development.

This is important to understanding the tendency toward resistance in the therapeutic situation as well. Essentially, the task of the psychoanalytic therapist is to help the client reduce the relative precision of the dominant response, and allowing an increased precision of the opposing response such that it can activate conscious-level inference, and thereby have greater flexibility in behavior. However, this is tantamount to a kind of “attack” on the attractor state of the generative model. The inertia of the present encoding of precisions as described above clarifies the intensity with which the client avoids this conflicting information in terms of actions taken by the person to prevent the progress of the therapeutic activity (Nord et al., 2017 have recently connected the vigor of avoidance activities with the predictions regarding the likelihood of catastrophe, providing interesting possibilities for a predictive coding-informed perspective on avoidance, and potentially therefore, resistance).

However, this resistance or inertia against psychological change is not only evident in the actions the person takes to prevent the therapeutic progress, but also the updating of the generative model in such a way that the new information is “explained away” in an intellectual sense. This may be linked to the common observation in therapy where the therapist's interpretation, rather than facilitating the client toward meaningful restructuring of their ego defenses, rather just becomes another link in the chain of the client's defenses. The client understands or may even agree with the therapist, but meaningful change does not take place. The client is able to generate new verbalizations and thought in response to the therapist's efforts that merely support the generative model rather than driving change. The present formulation describing the progressive increases in complexity of the “defensive” generative model helps make sense of this phenomenon as well.

## Some implications for psychoanalytic theory and therapy

A useful element of the formulation presented in this paper is that it appears to address the problems related to the “signal” theory of unpleasurable discharge that Freud developed in “The Interpretation of Dreams” 1990/1991. Here, Freud was pressed to explain how the psychic apparatus could prevent the mind from thinking of or remembering psychic material that caused unpleasurable discharge without experiencing it first. He suggested that there is a preliminary release of unpleasure associated with psychic activity that acts as a signal to the preconscious gate that discharge will cause unpleasure. As suggested in Grobbelaar (1989) and Connolly and van Deventer (2017), this process was never founded upon a suitable explanatory framework. However, the present formulation using expected free energy accomplishes this task. In essence, the updating of the generative model after the first experience of conflict means that the conflict state itself becomes reflected at a superordinate level of organization through the altered precisions. The sensory stimuli which would previously have generated the conflict state of uncertainty now generates the defense state that privileges one response over another. An example of such a response might be an inhibitory response of the prefrontal cortex toward the limbic system, which now occurs without necessarily reexperiencing the initial conflict state, but is rather the result of a downward prediction encoded at a cortical level. In essence the conflict is now “predicted” and “resolved” through one stroke, through the precision weightings toward one pole of the conflict now avoiding the uncertainty of the conflict state. Certainly, the organism also learns to avoid stimuli that activate that state or the surprise related to it. As stated earlier, this may be a reason why psychoanalytic conflict is difficult to measure in imaging of adult brains due to the fact that the established inhibitory, repressive behaviors of the brain, may often succeed in preventing the full experience of conflict as it has been defined in this paper.

A last implication that will be examined here relates to the role of therapy in restructuring the generative model. In one sense, the therapist could just point out to the client that there are actions that they are motivated to perform, though they aren't aware of it. Freud addressed such a situation in “Wild psycho-analysis” (1910) where he suggested that simply telling the client that they have unconscious motives are likely to make the client uncomfortable as it activates the conflict around it. He felt that such direct statements without regard to the therapeutic process brought psychoanalysis into disrepute as clients made so uncomfortable by comments such as this were often vocal in their condemnation of professionals who made such statements toward them, though Freud also felt that in the long run they might ultimately be helped by such statements as they drew the client's attention to the difficulty, at least. However, in that same paper, he suggested that a more therapeutically effective response (that also protected the dignity of the discipline) took into account two factors. Firstly, the readiness of the client, in the sense that they themselves were “in the neighborhood of” recognizing the repressed motivations themselves (which implies a lower FE cost in terms of perceiving it), but also that the relationship between therapist and client had reached a certain stage of emotional closeness in their relationship. This last is critical in the sense that the intensity or nature of the relationship with the therapist somehow alters the computation made by the person in terms of precisions related to expected free energy. An important question for future work is to state exactly *how* the relationship achieves this change that allows a recalculation of precisions associated with the conflict situation and the prototype emotions often associated with these.

One idea worth considering is the psychoanalytic notion of containment as articulated by Bion (1963). Based on Klein's (1946) concept, containment refers to the idea that the painful emotions and anxieties experienced by a person can in a sense be reduced in a relationship with another person, through a projection of the painful experience into the other who is experienced as becoming (through projective identification) the “bad” parts of the self. This seems to reduce the intensity of the emotions activated, and make the feelings seem more manageable. An example would be a person managing feelings of anxiety at separating from a loved one by (wrongly) perceiving a loved one as being very anxious about them instead, and feeling contempt for the other's perceived dependency. In this way the other person “contains” the feelings of anxiety. A precondition of this projective identification is the experience of the other as “good” in the sense that they can tolerate the negative emotions and be expected not to retaliate or abandon the person—in this sense the relationship is perceived as safe, despite these projective identifications of “bad” emotions. While the concept of containment from a FEP perspective requires a detailed treatment of its own, we could suggest that this perceived safety of the relationship must surely alter the perceived consequences of acting on emotions that might otherwise be repressed. Here we use again the example from Hopkins (2016) of the child who showed a pattern of fear responses (e.g., seeking reassurance) in key relationships while angry responses appeared absent, and developed into an adult who repressed angry responses in primary relationships. While the fear of expressing anger may have overwhelmed the young child, the adult in therapy who could feel anger toward a perceived abandonment by the “safe” therapist can learn to anticipate a far lower free energy cost of acting on that anger toward the therapist. This also forms the basis of Freud's (1912/1963) understanding of the therapeutic mechanism of transference, where the repressed emotion can be felt toward the therapist, allowing for it to achieve consciousness where it might otherwise not have. However, these remarks require more rigorous development in future than given here.

## Conclusion

The present paper has examined the Freudian notion of conflict, and assumed that this part of the theory requires a quantitative explanatory framework. After highlighting the failed explanation of Freud's energic theory, a formulation around expected free energy was shown to be a viable alternative to the energic theory. This formulation proposes a computational or mathematical formalization of conflict, which refers to the situation of relatively equivalent expected free energy of a number of actions under competing polices. This formulation also offers a formalization of defense as a recalibration of precisions at a hierarchically superordinate level of organization. This defensive organization is viewed as constraining the further development of the generative model, such that it maintains an attractor state characterized by the defensive operation, though it manifests in behavior in a complex and multi-faceted way. Implications of this formulation were explored, with the ongoing question of the role of the therapeutic relationship identified as an ongoing question.

The free energy principle and predictive coding presents an exciting opportunity to psychoanalysis, in that core conceptual foundations of psychoanalysis can be re-examined in the light of predictive coding, not only in order to demonstrate the viability of the basic theory of psychoanalysis relative to a foundation in systems theory and neuroscience, but also to consider how the theory may need to be recast in a newer systems-based language that makes these links. Although some way off at this stage, one of the practical utilities of having a formal theory is that one can simulate active inference and dyadic interactions. In principle, this makes it possible to create *in silico* psychotherapy and provide proof of principle of some of the dynamics that one might hypothesize. Such work may also eventually influence the clinical practice and training of psychoanalytic theory.

## Author contributions

The author confirms being the sole contributor of this work and approved it for publication.

### Conflict of interest statement

The author declares that the research was conducted in the absence of any commercial or financial relationships that could be construed as a potential conflict of interest.

## Footnotes

^1^The emotional complexity that is minimized here formally refers to the Kullback–Leibler divergence between posterior beliefs about policies or courses of action, relative to prior beliefs, which will be explained later in this paper.

^2^For a technical illustration of this sort of deep hierarchical inference see Friston et al. (2017b) which describes simulations dealing with the simple act of reading; where choosing which page to look at contextualizes, and informs choosing which sentence to sample, which contextualizes and informs, choosing the word to fixate on—and so on).
